# Knowledge, attitudes and practices on rift valley fever among pastoral and agropastoral communities of Ngorongoro in the rift valley ecosystem, Tanzania, conducted in 2021/2022

**DOI:** 10.1371/journal.pntd.0011560

**Published:** 2023-08-23

**Authors:** Amina Ramadhani Issae, Abdul Ahmed Selemani Katakweba, Rose Peter Kicheleri, Augustino Alfred Chengula, Christopher Jacob Kasanga

**Affiliations:** 1 African Centre of Excellence for Innovative Rodent Pest Management and Biosensor Technology Development, Sokoine University of Agriculture, Morogoro, Tanzania; 2 Department of Wildlife Management, Sokoine University of Agriculture, Morogoro, Tanzania; 3 Institute of Pest Management, Sokoine University of Agriculture, Morogoro, Tanzania; 4 Department of Veterinary Microbiology, Parasitology and Biotechnology, Sokoine University of Agriculture, Morogoro, Tanzania; University of Pittsburgh School of Medicine, UNITED STATES

## Abstract

Epidemics of Rift Valley fever (RVF), a mosquito-borne zoonotic disease caused by RVF virus, have been linked to exceptionally heavy rainfall and widespread flooding. The disease is endemic in most African countries and pose a major global health risk. Given that the disease was reported in various districts of Tanzania, we hypothesized a lack of knowledge about RVF epidemiology among agropastoral and pastoral communities. The research took place in a period of 7 months, from July, 2021 to January, 2022. The aim of this study was to assess the knowledge, attitudes, and practices (KAP) among the agropastoral and pastoral communities of Ngorongoro district towards RVF. The survey employed a mixed method system, which included 3 focus groups (each comprised 12 individuals), 20 key informant interviews and administration of questionnaire (N = 352) in agropastoral and pastoral community members of Ngorongoro district. The relationship between demographic characteristics and communities’ knowledge, attitudes, and practices regarding RVF was observed using a multiple logistic regression model. A total of 352 participants were interviewed, with the majority (67.61%) being male and 32.39% being female, majority (39.5%) attending primary school, and majority (58.2%) being pastoralists. The findings showed that only 36.1%, 38.64% and 16.19% of participants had good knowledge, positive attitude and good practices regarding RVF respectively. Significant demographic factors related with knowledge included: gender (OR = 1.9, CI = 1.03–3.56, P = 0.041), education levels (primary: OR = 3.97, CI = 2–8.16, P = 0.000; secondary: OR = 15.27, CI = 5.5–46.23, P = 0.000 and college: OR = 34. 23, CI = 5.4–67.22, P = 0.000), and locality (Pinyinyi: OR = 0.14, CI = 0.05–0.38, P = 0.000 and Sale: OR = 0.14, CI = 0.04–0.44, P = 0.001). Male participants showed significant positive attitude towards RVF compared to female (OR = 2.37, CI = 1.35–4.17, P = 0.003). Individuals with formal education showed a significant positive attitude toward RVF compared to informal (OR>1, P<0.05). Agropastoral members showed a significant negative attitude toward RVF compared to pastoralists (OR = 0.51, CI = 0.26–0.99, P = 0.048). The calculated RVF prevention practices values were insignificantly (P = 0.853) correlated with knowledge values. The significant correlation between knowledge and attitude, as well as attitude and practice were found (P<0.05). In general, the study revealed poor knowledge, negative attitude and poor practices of communities towards RVF. The lack of regular education programs to make the communities aware of the disease was implicated for these findings. This recommends that provision of health education should be a long-term practice among agropastoral and pastoral communities in order to prevent further RVF outbreaks in Tanzania.

## Author summary

In the current study, we demonstrated that the KAP for RVF transmission and prevention is low among the pastoral and agropastoral community members in Ngorongoro district, Tanzania. The causes, route of transmission and signs of RVF in both humans and animals were mostly unknown to the respondents. The population was unaware that the high mortality rate of young animals and the miscarriage of pregnant animals were severe signs of RVF. The crucial preventive measures against RVF were not being used, which created a gap between recommended and real practices. Low level of knowledge about the RVF was linked to poor preventive efforts, so raising awareness could launch public awareness in a positive way. We suggest that important authorities should launch public awareness campaigns to help bridge these knowledge gaps and strengthen people’s understanding about RVF. It might be necessary to use radio and mobile applications to provide the public with access to such health information. In order to alter behavioral patterns in the study communities to avert RVF occurrences, barriers to change must be identified and eliminated. The livelihoods of agropastoral and pastoralist communities will benefit if these procedures are put into action since they may prevent regular disruptions of livestock market chains that occur whenever RVF outbreaks happen.

## Introduction

Rift Valley fever (RVF) is an ecologically complex arthropod-borne viral zoonotic disease caused by RVF virus, which belongs to order *Bunyavirales*, family *Phenuiviridae*, genus *Phlebovirus* [1]. The disease was originally documented in 1930 following a storm of abortions and sudden deaths in sheep along the coasts of Lake Naivasha in Kenya’s greater Rift Valley [2]. Following infected livestock trade from the horn of Africa, RVF spread in September 2000 to Saudi Arabia and Yemen [3,4]. The largest outbreak in East Africa occurred in 1997–1998 and 2006–2007, which encompassed Kenya, Somalia, and Tanzania, with over 1000 confirmed positive cases and 300 deaths [5,6]. Between 1947 and 1978, four Tanzanian districts (Ngorongoro, Simanjiro, Monduli and Hai) experienced epidemics, as well as further outbreaks from1997 to 2007.

The RVF virus is maintained in infected mosquitoes and transmitted to animals via mosquito bites. Mosquito species in the genera *Aedes*, *Anopheles*, *Culex*, *Eretmapodites*, *Coquilletidia* and *Mansonia* have been associated with RVF virus transmission [7]. These vectors have been found in various regions throughout Africa [8]. Transmission of RVF virus by hematophagous (blood-feeding) flies is also possible [9]. In 1997–98, a major outbreak occurred in Kenya, Somalia, and Tanzania following an El Niño event causing extensive flooding [10]. Flooding triggers the hatching of eggs of *Aedes* mosquito species, which are the RVF virus’s main reservoirs. According to studies conducted in various locations, the RVF virus is transmitted to domestic animals either through mosquito bites or direct contact with infected tissues or body fluids, particularly when abortions are involved [10].

The majority of human infections result from direct or indirect contact with the blood or organs of infected animals [10,11]. Human infections have also resulted from the bites of infected mosquitoes, most commonly the *Aedes* and *Culex* mosquitoes. The female mosquito is also capable of transmitting the virus directly to her offspring via eggs (transovarial) leading to new generations of infected mosquitoes hatching from eggs [10,11]. Consuming raw meat and milk, caring of sick animals, assisting parturition, and sheltering livestock inside residential houses are all reported risk factors for RVF virus infection in humans [11]. Affected animals, both clinically and sub clinically, are an important source of infection to humans; to date, no human-to-human transmission has been documented [12].

The RVF virus can cause mild to fatal illness in wildlife, domestic animals, and humans [12]. Studies reported high morbidity and mortality approaching 100% in lambs and kids of less than 12 weeks of age [5,6,13]. Hemorrhagic fever, mucopurulent nasal discharge, abortions, salivation, anorexia, fetid diarrhea, and uncoordinated gait are some of the symptoms in infected animals [5,6,13]. In humans, RVF virus can induce flu-like symptoms that can progress to a severe disease characterized by hemorrhagic fever and encephalitis, with a 1–5 percent fatality rate [14]. For a successful early warning monitoring system to respond to outbreaks in a timely manner, the communities must have appropriate knowledge of how to identify RVF indications in advance [14].

The socio-economic consequences of the RVF include animal and human fatalities, disruption of livestock market chains, human hunger, malnutrition, and monetary losses at the individual and national levels [15]. As a result of its socioeconomic implications, both veterinary and public health officials in Tanzania are concerned about the disease [15].

Apart from the disease’s socioeconomic impact in Tanzania, just one study was undertaken to assess communities’ knowledge about the RVF infections [16]. Because communities’ knowledge and attitudes are associated with diseases management, Knowledge, Attitude and Practices (KAP) research is becoming increasingly important in infection prevention and control efforts [16,17]. Also, provided that RVF had occurred in several districts in Tanzania, we hypothesized a lack of sufficient knowledge about RVF epidemiology among agropastoral and pastoral communities. Therefore, the goal of a recent study was to identify and contribute to bridging information gaps by examining the level of knowledge, attitudes, and practices of agropastoral and pastoral communities in Ngorongoro district about RVF. The findings will contribute in building effective and long-term effective RVF prevention efforts. This would benefit pastoral and agropastoral societies whose livelihoods have been threatened by RVF outbreaks since they rely on the sale of livestock and livestock products.

## Materials and methods

### Ethics statement

The protocol to do this study was reviewed by Ethical Review Committee of the National Institute for Medical Research (NIMR) (Ref. No. NIMR/HQ/R.8a/Vol. IX/3676; 19^th^ May, 2021). Also, Sokoine University of Agriculture provided the permission letter for conducting this study (Ref. No. SUA/ADM/R.1/8A/718; 3^rd^ February, 2021). Moreover, the local administrative authorities of Arusha region (Ref. No. FA.132/95/01/38; 12^th^ February, 2021) and Ngorongoro district (Ref. No. AB.114/354/01/134; 1^st^ April, 2021) also gave permission. Prior to the start of face-to-face interviews, participant provided written informed consent. In cases where the participant was unable to write and read, verbal assent was obtained.

### Description of the study area

The proposed study was conducted in five villages (Orgosorok, Malambo, Sale, Pinyinyi and Engarasero) of Ngorongoro district where animal and human cases of RVF were reported in the previous outbreaks [18]. The district is in the eastern Rift Valley ecosystem that suffered the outbreaks from 1947 to 1978 and 2006/2007 [18]. Ngorongoro District is one of the seven districts of the Arusha Region of Tanzania. It is bordered to the east by Monduli District, to the south by the Karatu District and to the west by the Mara Region. The district has an area of about 14,036 square kilometers located between latitudes 30.30’south of the equator and longitudes of 35.42 ‘east of Greenwich and is between 1,009 and 3,645 meters above sea level [18]. According to the 2012 Tanzania National Census, the population of the Ngorongoro district was 174,278 [19]. Administratively, the district is divided into three divisions (Ngorongoro, Loliondo and Sale) and 20 Wards. The district has a moderate temperature and tropical climate with average rainfall of 800 mm to 1,000 mm [19]. The major ethnic tribes are Masai and Sonjo, who depends on livestock keeping and sometimes on crop farming for their livelihoods [20]. The district is characterized by low undulating plains with low-lying altitude [20]. The main vegetation in the study area is shrubs of acacia species and grass; open and thick forests [20].

### Study design and sampling procedures

A cross-sectional study was done in Ngorongoro district to investigate RVF knowledge, attitudes and practices among agropastoral and pastoral residents. The study population comprised of all households in selected villages and the sampling frame was the list of the households in each village. Villages were purposefully selected based on the following criteria: population density, accessibility of the area, located at Great Rift Valley, livestock and wild animal availability. The study was conducted in five villages namely Orgosorok, Malambo, Sale, Engarasero and Pinyinyi. Purposeful sampling was also used in the selection of households based on availability of animals like ruminants, dogs and rodents. At household level, the written consent of the household head was sought before the interview. The household head or any other resident person (18 years and above) was interviewed based on the assumptions like knowledge on diseases and ability of deciding on their participation in the study.

### Sample size determination

This formula $n=\frac{Z^{2}p(1-P)}{d^{2}}$ was used for estimation of the households’ sample size [21], where: n = sample size estimate, Z = 1.96; standard normal variety at 5% error **(**P<0.05**)**, and assuming a response distribution of 50%. **(**1-p**)** = the probability of knowledge and d = absolute error or precision (5%). The calculated household sample size (n) is 384. By considering the 5% mark up for missing samples; the infinite population size becomes 403. Then, the sample size adjusted to finite population (n_c_) was computed using the equation; n_c_ = n/ (1+f) where; n = estimated sample size (403) and f = sampling fraction (403/14,195), this gave 392 number of households however only 352 households were interviewed in Ngorongoro district due to various challenges encountered during field work (unwillingness of individuals, difficult accessibilities of areas and reallocation of pastoralists). However, the 352 households interviewed had a statistical power of 0.9 value. This indicated that the results are statistically significant by 90% which is scientifically acceptable. The proportionate formula was applied (N = HW1 + HW2+ HW3) to determine the number of households to be interviewed in each village, where: N = total number of households interviewed in the district and HW1 + HW2+ HW3 = number of households in the selected village. Selection of hamlets in each village was done purposefully based on history of RVF cases and availability of animals as well as areas of humans-wildlife interface (word of mouth from the villages leaders).

### Data collection

The researchers explained the study’s purpose to all respondents, who included the local communities’ leaders and government officials. Before being interviewed, participants gave written or oral consent to participate in the study. All collected data and information were confidentially handled and stored.

#### Questionnaire survey

Based on literature review of Rift Valley fever, a semi-structured questionnaire tool was established [22]. The survey was divided into five categories: (i) respondent demographics; (ii) household characteristics (iii) knowledge on RVF virus vectors, transmission modes in humans and animals, and clinical signs; (iii) attitude toward RVF management and (v) practices against prevention of RVF. To ensure that questions were appropriate, the questionnaire was pretested in 20 households and amended according to the problems recognized. The questionnaire was filled by visiting one house after another in each study village. Enumerators who were fluent in English and Swahili languages were deployed in administration of the questionnaire. Questions were asked in Swahili and responses recorded in English to serve time for retranslation. Investigators kept a close eye on the questionnaire administration process and double-checked the completed form to ensure that the acquired data was of high quality.

#### Interviews with the key informants (KIs)

In the Ngorongoro district, a total of twenty KI interviews were done to collect more information about Rift Valley fever. The study employed a targeted sample technique, and participation was entirely voluntary. Local collaborators at each study ward/village identified participants; four individuals per village. The community health workers, nurses, clinical officers, assistant field livestock/ livestock officers, veterinary/medical officers and elected local authority leaders were among those who participated in the interview. Participants were asked on the RVF etiology, transmission, symptoms, prevention, socioeconomic implications, risk factors and dissemination of education among communities. The interviews were conducted by a trained researcher, and notes were collected by the note takers. The interviews were also recorded using a phone recorder and the audio records were transcribed.

#### Focus group discussions (FGD)

We conducted three FGD and each group comprised 12 respondents [22]. Purposeful sampling was used in selecting participants for the FGD, targeting on adults (aged 18years and above) with confidence in talking, permanent residents, animals’ keeper and person who didn’t participate in questionnaire. The FGD was facilitated by a trained community health worker or livestock officer together with the researcher and there were two note takers. A semi-structured FGD guide was created and used during discussions. FGD participants were asked on transmission of RVF virus, signs of RVF in both humans and animals, control and prevention, risk factors of RVF and socioeconomic impact of RVF in the communities.

### Data analysis

#### Quantitative data analysis

For each question in the knowledge part, scores ranging from 1 to 5 were given to correct responses based on the question type. Furthermore, incorrect and don’t know responses were assigned zero scores. A knowledge score for each respondent was calculated by summing the number of correct answers out of the total scores [23]. Correct answers were assigned a score of 1, and answers that were incorrect, or selected as “I don’t know”, were assigned a score of 0 [23].

Attitudes regarding RVF prevention and control measures were assessed using the Likert scale system, with responses ranging from 1 to 5, with 1 = completely disagree; 2 = disagree; 3 = neutral; 4 = agree and 5 = completely agree [22,24]. During cross tabulations analysis, responses were dichotomized into (1) completely disagree/disagree/neutral and (2) completely agree/agree. Correspondents who completely agreed or agreed were reported to have positive attitudes toward RVF occurrence, while the rest were considered to have negative sentiments [22]. In the Practices section, each correspondent’s score was calculated by adding the number of correct responses to the nine questions posed.

The variables in the data were coded for easy entry and analysis. Data were entered into Microsoft Excel 2010 and edited to remove invalid variables and thereafter, exported to R software version 4.1.0 (2021) for analysis. Findings were presented in descriptive statistics like means, proportions and frequencies. Comparison of proportions for categorical variables such as socio-demographics, knowledge and practices were conducted by deploying the Chi-square test. The outcome variables were knowledge about RVF and preventive practices of RVF. Relationship between various factors and knowledge on RVF, attitude or practices on taking into account about the influence was assessed using multiple logistic regression. Odd ratios (ORs) and their corresponding’s 95% confidence interval (CIs) were calculated and P<0.05 was considered significant for all statistical analyses.

#### Qualitative data analysis

A deductive analysis approach was used for qualitative data collected from FDG and Key Informants interviews [25], whereas the researcher developed themes from the RVF literature review. FGD and Key Informants information was summarized manually according to themes presented in the discussions and interviews. Results are described in the text together with relevant speech marks.

## Results

### Quantitative data from questionnaire

#### Demographic characteristics of the studied communities

In the Ngorongoro district, a total of 352 personnel took part in the survey, with the majority of them being men (n = 238, 67.61%). Their median age group was 25–34 years old and the age ranged from 18–65 years. The majority of the population went to primary school (n = 139, 39.5%). Also, the majority (n = 205, 58.2%) were pastoralists (Table 1).

**Table 1:** Respondents demographic characteristics in Ngorongoro district.

Variable	Female n (%)	Male n (%)	Total N (%)
**Age group**			
Youth (18–34 years)	57(16.2)	89(25.3)	146 (41.5)
Adult (35–64 years)	54(15.3)	138(39.2)	192(54.5)
Elderly (≥65 years and above)	3(0.9)	11(3.1)	14(4.0)
**Level of education**			
Not attended school	58(16.5)	71(20.1)	129(36.6)
Did not completed school	7(2.0)	23(6.5)	30(8.5)
Primary	40(11.4)	99(21.1)	139(39.5)
Secondary	8(2.3)	35(10.0)	43(12.3)
College	1(0.3)	10(2.8)	11(3.1)
**Religion**			
Christian	84 (24.0)	165 (47.0)	249 (71.0)
Muslim	2 (1.0)	15 (4.0)	17 (5.0)
Traditional	28 (8.0)	58 (16)	86 (24.0)
**Marital status**			
Single	8(2.3)	15(4.2)	23(6.5)
Married	106(30.1)	223(63.4)	329(93.5)
**Occupation**			
Agropastoral	53(15.1)	94(26.7)	147(41.8)
Pastoralist	61(17.3)	144(40.9)	205(58.2)
**Locality (Villages)**			
Orgosorok	37(10.5)	74(21.0)	111(31.5)
Malambo	16(4.5)	38(10.8)	54(15.3)
Sale	27(7.7)	64(18.2)	91(25.9)
Engarasero	15(4.3)	30(8.5)	45(12.8)
Pinyinyi	19(5.5)	32(9.0)	51(14.5)
Total number of respondents	**114 (32.39)**	**238(67.61)**	**352(100)**

#### Household characteristics

Among 352 households visited during this study, 349 (99.15%) were headed by men and 3 were (0.85) headed by woman. A high proportion (48%) of the household size was found to be 1–5 members in each household in Ngorongoro district. About 35.2% of respondents own houses constructed by using animal feces (Table 2). Furthermore, 41.2% of houses have open windows and grasses were found to be the preferred thatching materials of roofs. Lastly, the results show that about 53.4% of households use a pit latrine. With the exception of toilets type, other variables were highly significant different (Table 2).

**Table 2:** Respondents household characteristics based on the study villages.

Variable	Engarasero n (%)	Orgosorok, n (%)	Malambo n (%)	Pinyinyi n (%)	Sale n (%)	Total N (%)	χ^2^ tests	P-value
**Household size**							64.915	<0.0001
1–5	20(11.8)	71(42.0)	30(17.8)	19(11.2)	29(17.2)	169(48.0)		
6–10	17(12.7)	33(24.6)	13(9.7)	21(15.7)	50(37.3)	134(38.1)		
Above 10	8 (16.3)	7 (14.3)	11 (22.4)	11(22.4)	12(24.5)	49(13.9)		
**House walls**							171.27	<0.0001
Block	1 (2.1)	23(47.9)	5(10.4)	2(4.2)	17(35.4)	48 (13.6)		
Block with concrete	0(0.0)	9(75.0)	0(0.0)	3(25.0)	0(0.0)	12(3.4)		
Mud	0(0.0)	48(28.6)	1(0.6)	46(287.4)	73(43.5)	168(47.7)		
Animal feces	44(35.5)	31(25.0)	48(38.7)	0(0.0)	1(0.8)	124 (35.3)		
**Type of window**							169.86	<0.0001
No window	24(15.7)	43(28.1)	15(9.8)	9(5.9)	62(40.5)	153(43.5)		
Open window	14(9.7)	44(30.3)	35(24.1)	32(22.1)	20(13.8)	145(41.2)		
Net window	5(21.7)	9(39.1)	1(4.3)	3(13.0)	5(21.7)	23(6.5)		
Window with shutters	2(6.5)	15(40.4)	3(9.7)	7(22.6)	4(12.9)	31(8.8)		
**Roof of the house**							118.39	<0.0001
Grass	33(16.8)	42(21.4)	38(19.4)	28(14.3)	55(28.1)	196(55.7)		
Mud and grass	0(0.0)	27(90.0)	3(10.0)	0(0.0)	0(0.0)	30(8.5)		
Iron sheet	12(9.5)	42(33.3)	13(10.3)	23(18.3)	36(28.6)	126(35.8)		
**Source of electricity**							235.57	<0.0001
Neither	38(16.0)	67(28.3)	38(16.0)	38(16.0)	56(23.6)	237(67.3)		
Solar	7(6.2)	42(37.2)	16(14.2)	13(11.5)	35(31.0)	113(32.1)		
TANESCO*	0(0.0)	2(100.0)	0(0.0)	0(0.0)	0(0.0)	2(0.6)		
**Type of toilets**							1.636	0.2008
No toilet	24(14.6)	27(16.5)	31(18.9)	6(3.7)	76(46.3)	164(46.6)		
Pit Latrine	21(11.2)	84(44.7)	23(12.2)	45(23.9)	15(8.0)	188(53.4)		

TANESCO = Tanzania Electric Supply Company Limited

#### Sources of various Information among agropastoral and pastoral communities

The study found that communities in the study area prefer use of phone (42.8%) and radio (29%) as the major source of communication (Table 3).

**Table 3:** Source of information dissemination among study villages in Ngorongoro district.

Variable	Engarasero n (%)	Orgosorok n (%)	Malambo n (%)	Pinyinyi n (%)	Sale n (%)	Total N (%)	χ^2^ tests	P-value
**Source of information**							289.45	P<0.0001
Gatherings	12(23.5)	15(29.4)	8(15.7)	3(5.9)	13(25.5)	51(13.3)		
Health servants	3(14.3)	8(38.1)	7(33.3)	0(0.0)	3(14.3)	21(5.5)		
Veterinary servants	3(14.3)	8(38.1)	7(33.3)	0(0.0)	3(14.3)	21(5.5)		
Phone	22(13.4)	46(28.0)	25(15.2)	26(15.9)	45(27.4)	164(42.8)		
Radio	6(5.4)	44(39.6)	17(15.3)	18(16.2)	26(23.4)	111(29.0)		
TV	3(20.0)	2(13.3)	0(0.0)	4(26.7)	6(40.0)	15(3.9)		

#### Knowledge about mosquitoes management and their zoonoses

The analysis of the knowledge score indicated that scores of respondents ranged from 0 to 13 out of a total of 17 total points. Among the participants, only 36.1% (n = 127) managed to get above 50% of the total scores, showing poor knowledge of the community. The most known mosquito borne disease is Malaria as stated by 89.22% of respondents. 88.35% of participants reported the use of bed nets as the main preventive measure of mosquito bites (S1 Table).

#### Relationship between respondent factors and good knowledge about mosquitoes

Most of the respondents’ demographic factors had insignificant positive influence on knowledge about mosquito-borne zoonosis and control measures (P>0.05). Only education level was found to have significant positive influence on knowledge about mosquito borne diseases as well as on control measures (P<0.05), as indicated in Table 4.

**Table 4:** Multiple logistic regression analysis of the relationship between demographic factors and good knowledge about Mosquitoes borne diseases.

Variable	OR	Confidence interval (CI, 95%)	P-value
**Sex**			
Female	Reference		
Male	1.15	0.58–2.28	0.693
**Age**			
Elderly	Reference		
Adult	1.17	0.22–6.32	0.859
Youth	1.08	0.19–6.11	0.092
**Level of education**			
Not attended school	Reference		
Did not completed school	1.62	0.39–6.86	0.509
Primary	3.55	1.51–8.38	0.004**
Secondary	9.02	3.15–25.85	0.000***
College	17.32	3.82–78.51	0.000***
**Occupation**			
Pastoralist	Reference		
Agropastoral	0.61	0.29–1.28	0.191
**Religion**			
Muslim	Reference		
Christian	1.96	0.22–16.76	0.550
Traditional	3.48	0.39–31.13	0.265
**Marital status**			
Single	Reference		
Married	0.91	0.28–2.91	0.872
**Ward (locality)**			
Engarasero	Reference		
Malambo	0.60	0.19–1.83	0.368
Pinyinyi	1.43	0.51–4.06	0.497
Sale	0.86	0.27–2.73	0.801
Orgosorok	1.03	0.41–2.55	0.951

* = Significant at P< 0.05.

** = Moderately significant at P <0.005,

*** = Highly significant at P <0.0001

#### Knowledge of the communities about rift valley fever symptoms and transmission

The analysis of knowledge score demonstrated that out of total points (17), respondents score ranged from 0 to 14 points. Whereby, only 37 respondents equivalent to 10.51% scored above the average of total score, implying low level of knowledge of the communities. Among 352 total participants, 52% respondents had heard about RVF and 25% of them knew that the disease is zoonotic (S2 Table). 45.74% of respondents (n = 161) mentioned ruminants as the group of animals that can succumb from RVF infection. Considering signs of RVF infection in humans, febrile fever 13.35% followed by headache 10.23% symptoms were frequently mentioned. In regard to signs of RVF in animals, 30.4% and 17.61% participants mentioned fever followed by abortion as major signs of RVF, respectively. Mosquito bites as the significant known route of RVFV transmission in animals was mentioned by 13.64% of respondents (S2 Table).

#### Relationship between demographic factors and good knowledge about RVF

The results of this study show that the communities’ knowledge on RVF is significantly related to demographic characteristics like sex, level of education and location (P<0.05) as presented in Table 5 below. Males, respondents with primary, secondary and college education demonstrated significant (P<0.05) high knowledge, while inhabitants of Pinyinyi and Sale were found to have significant (P<0.05) less knowledge about RVF compared to those of Engarasero (Table 5).

**Table 5:** Multiple logistic regression analysis of the relationship among demographic factors and good knowledge on RVF signs and transmission.

Variable	OR	Confidence interval (95%)	P-value
**Sex**			
Female	Reference		
Male	1.90	1.03–3.56	0.041*
**Age**			
Elderly	Reference		
Adult	0.77	0.22–2.93	0.686
Youth	0.45	0.12–1.80	0.239
**Level of education**			
Not attended school	Reference		
Did not completed school	1.05	0.21–4.01	0.944
Primary	3.97	2.00–8.16	0.000***
Secondary	15.27	5.50–46.23	0.000***
College	34.23	5.40–67.22	0.001***
**Occupation**			
Pastoralist	Reference		
Agropastoral	1.72	0.85–3.52	0.135
**Religion**			
Muslim	Reference		
Christian	1.02	0.20–7.73	0.987
Traditional	0.72	0.14–5.63	0.724
**Marital status**			
Single	Reference		
Married	2.23	0.67–8.23	0.205
**Villages (locality)**			
Engarasero	Reference		
Malambo	0.47	0.18–1.20	0.118
Pinyinyi	0.14	0.05–0.38	0.000***
Sale	0.14	0.04–0.44	0.001***
Orgosorok	0.95	0.39–2.27	0.914

* = Significant at P< 0.05.

** and *** = Highly significant at P <0.0001

#### Communities’ attitude toward RVF transmission and mosquitoes’ management

The results indicate that among 352 respondents, 269 (76.42%) and 83 (23.35%) showed positive and negative attitude toward mosquito-borne diseases, respectively. The majority of respondents agreed that mosquitoes have adverse effects on their quality of life and they are at risk of getting diseases through mosquito bites (S3 Table).

#### Relationship between factors and attitude toward mosquito borne diseases

The results of the logistic regression model indicated that sex, level of education, marital status and locality have significant (P<0.05) influence on attitude toward mosquitoes borne diseases and management (Table 6). Male respondents showed more positive attitude compared to female one and the difference was significant (P = 0.006). Individuals with primary and secondary level of education were found to have a more positive attitude compared to those didn’t attend school, and this difference was statistically significant (P<0.05).

**Table 6:** Multiple logistic regression analysis of the relationship between demographic factors and positive attitude toward Mosquito borne diseases.

Variable	OR	Confidence interval (CI, 95%)	P-value
**Sex**			
Female	Reference		
Male	2.29	1.26–4.14	0.006**
**Age**			
Elderly	Reference		
Adult	1.02	0.25–4.11	0.981
Youth	0.93	0.22–3.95	0.917
**Level of education**			
Not attended school	Reference		
Did not completed school	1.51	0.53–4.34	0.444
Primary	2.44	1.21–4.91	0.013*
Secondary	5.57	1.61–19.26	0.007**
College	1.36e+07	0.00-Inf	0.981
**Occupation**			
Pastoralist	Reference		
Agropastoral	0.66	0.31–1.41	0.289
**Religion**			
Muslim	Reference		
Christian	0.34	0.06–1.81	0.206
Traditional	0.31	0.06–1.59	0.159
**Marital status**			
Single	Reference		
Married	3.41	1.14–10.17	0.028*
**Villages (locality)**			
Engarasero	Reference		
Malambo	0.53	0.15–1.70	0.270
Pinyinyi	8.65	0.89–83.39	0.062
Sale	0.44	0.12–1.58	0.207
Orgosorok	0.22	0.07–0.65	0.006**

* = Significant at P< 0.05. and

** = Moderately significant at P <0.005.

#### Communities’ attitudes toward Rift Valley Fever transmission and prevention

Among 352 participants, 136 (38.64%) and 216 (61.36%) respondents showed positive and negative attitudes, respectively. The findings showed that most of participants neither “agrees” nor “disagrees” about the statements posed to them about RVF. The majority of respondents didn’t decide on RVF being a hazardous disease of public health importance. The majority of respondents didn’t decide on being at risk of getting RVF virus infection. The majority of them didn.t decide on wearing protective gears can prevent transmission of RVF virus, and that the interaction between humans and animals can facilitate RVF transmission (S4 Table)

#### Relationship between demographic factors and attitudes toward RVF transmission

The results indicated that sex, education and occupation have a significant (P<0.05) influence on communities’ attitudes toward RVF transmission and prevention. Males have a significant (P = 0.003) positive influence on attitude towards RVF. Also, individuals who attended school found to have a significant (P,0.05) positive influence on attitude toward RVF transmission and prevention (Table 7). Moreover, agropastoral were found to have a significant (P = 0.048) negative influence on attitude toward RVF.

**Table 7:** Multiple logistic regression analysis of the relationship between demographic factors and positive attitude toward RVF transmission and prevention.

Variable	OR	Confidence interval (CI, 95%)	P-value
**Sex**			
Female	Reference		
Male	2.37	1.35–4.17	0.003**
**Age**			
Elderly	Reference		
Adult	0.49	0.15–1.63	0.245
Youth	0.37	0.11–1.31	0.125
**Level of education**			
Not attended school	Reference		
Did not completed school	2.89	1.12–7.43	0.027*
Primary	2.42	1.31–4.47	0.005**
Secondary	4.15	1.74–9.88	0.001**
College	46.25	5.19–411.7	0.001**
**Occupation**			
Pastoralist	Reference		
Agropastoral	0.51	0.26–0.99	0.048*
**Religion**			
Muslim	Reference		
Christian	0.43	0.13–1.45	0.174
Traditional	0.35	0.11–1.16	0.087
**Marital status**			
Single	Reference		
Married	2.70	0.85–8.62	0.094
**Villages (locality)**			
Engarasero	Reference		
Malambo	0.53	0.22–1.37	0.196
Pinyinyi	1.03	0.40–2.60	0.957
Sale	1.25	0.46–3.41	0.668
Orgosorok	0.83	0.37–1.88	0.661

* = Significant at P< 0.05. and ** = Moderately significant at P <0.005

#### Communities’ practices on RVF transmission and prevention

The analysis of participants practices was characterized as effective infection prevention practices or non-effective infection prevention practices (effective when the score was above 50% and non-effective when the score was below the 50% of total score). The maximum score was 11, and 57 (16.19%) of the 352 respondents showed effective infection prevention practices. The majority of participants (n = 336 or 95.5%) do not avoid contacting sick or dead animals’ fluids or handling aborted fetuses with their bare hands (S5 Table). 60.23% drink fresh raw animal blood and 77.27% keep animals in their sleeping area (S5 Table). Bed netting, insecticide sprays, and repellent ointments, as well as the treatment of stagnant water, were reported as being used in the communities to manage mosquito populations and prevent mosquito bites. However, the majority (35.51%) of respondents reported the use of bed nets as the preferred control measure against mosquito bites (S5 Table).

#### Multiple logistic regression analysis of factors and preventive practices of RVF

Concerning relationships among demographic characteristics and effective preventive practice of RVF, the performed logistic regression model indicated that respondents with primary and secondary education levels had significantly (P = 0.018) positive influence on preventive practices (Table 8). On other the other hand, individuals of Malambo and Engarasero had significant (P<0.05) negative influence on effective preventive measures of RVF transmission compared to those of Orgosorok (Table 8). While those of Sale village had significant (P = 0.000) positive influence on preventive practices of RVF compared to Orgosorok’s respondents. Moreover, pastoralists had significant (P = 0.005) positive influence on effective preventive practices of RVF than agropastoral community (Table 8).

**Table 8:** Multiple logistic regression analysis of the relationship between demographic factors and effective preventive practices of RVF.

Variable	OR	Confidence interval (CI, 95%)	P-value
**Sex**			
Female	Reference		
Male	0.79	0.46–1.37	0.408
**Age**			
Elderly	Reference		
Adult	1.41	0.41–4.81	0.586
Youth	1.48	0.41–5.30	0.549
**Level of education**			
Not attended school	Reference		
Did not completed school	2.74	0.92–8.11	0.069
Primary	2.43	1.33–4.44	0.004**
Secondary	4.12	1.60–10.59	0.003**
College	2.99	0.72–12.45	0.131
**Occupation**			
Agropastoral	Reference		
Pastoralist	2.53	1.31–4.87	0.005**
**Religion**			
Muslim	Reference		
Christian	1.86	0.46–7.51	0.383
Traditional	1.62	0.42–6.29	0.483
**Marital status**			
Single	Reference		
Married	0.46	0.15–1.44	0.185
**Villages (locality)**			
Orgosorok	Reference		
Malambo	0.47	0.22–0.98	0.043*
Pinyinyi	1.89	0.85–4.16	0.116
Sale	6.02	2.40–15.07	0.000***
Engarasero	0.28	0.12–0.62	0.002**

* = Significant at P< 0.05.

** = Moderately significant at P <0.005,

*** = Highly significant at P <0.0001

#### Correlation between knowledge, attitude and practice

Results of this study indicated that knowledge about RVF was insignificantly (P = 0.853) negatively correlated with RVF effective preventive practices. Also, results indicates that there is a significant relationship between knowledge and attitude, as well as attitude and practice (P<0.05) as shown in Table 9.

**Table 9:** Correlation of total knowledge score, total attitude score and total practice responses score of respondents about RVF disease.

Scores	Mean	SD	N	Correlation coefficient	P- value
Total knowledge score	2.912	2.656	352	-0.009	0.853
Total practice score	4.821	1.099			
Total knowledge score	2.912	2.656	352	0.545	0.000***
Total attitude score	3.716	3.531			
Total attitude score	3.716	3.531	352	0.162	0.002**
Total practice score	4.821	1.099			

** = Moderately significant at P <0.005,

*** = Highly significant at P <0.0001

### Qualitative information from KI Interviews and FGD

#### Health education delivery system to the communities in Ngorongoro district

During the Key Informant (KI) Interviews, the participants were probed on how the communities get information about diseases and preventive measures to adopt. Health workers often conduct health education campaigns at the community level depending on availability of funds (Key Informants). Health workers deliver health education to the community level but with difficulties due to inadequate facilitation from the government (Key Informants). Health workers in Pinyinyi and Sale villages usually have a regular weekly and monthly outreach training at the community level (Key Informants). They provide health education to individuals or patients who attend clinics in working facilities like dispensaries and health centers or hospitals. They give education to targeted groups of people like pregnant women. Therefore, the provision of health education depends on government’s priorities and the availabilities of funds (Key Informants).

#### Livestock vaccination program in Ngorongoro district

In the last RVF episode in 2007, the government conducted vaccination of small ruminants in the district. The local authority and ministry of livestock together organized the vaccination program in various places of RVF outbreaks (Key informants). The interviews with participants indicated that the intervention was carried out late after livestock acquired the infection (FGD members). Vaccination process to small ruminants was also performed in 2011 and 2012 in RVF outbreak prone villages (Key informants).

#### Socio-economic impact of RVF in the communities of Ngorongoro district

During the FGD and key informants’ interviews, participants described several socio-economic impacts as demonstrated in the following quotes: “During the outbreak, there were high mortality of sheep and goats along the Rift Valley ecosystem in villages like Malambo, Engarasero and Pinyinyi (Key informants). Also, there was a deficiency of food particularly to the pastoralists community that mostly depends on meat and milk diets (FGD respondent). Furthermore, there was lack of income as the government closed the livestock markets and restricted animals’ movement (KIs and FGD). During outbreaks, approximately 35 individuals were suspected of dying from RVF infection. These cases involved the livestock keepers who consumed sick or dead animals in their households (Key informants).

#### Socio-cultural behaviors of the agropastoral and pastoralists of Ngorongoro

Several cultural behaviors were perceived in this study as described by FGD and KIs respondents during the discussions and interviews. The risk behaviors included drinking raw milk and blood, consumption of raw meat (kidneys and livers), sheltering of young animals in human houses, handling of aborted fetuses with bare hands and the use of animal feces for construction of human houses. However, some of the respondents stated that some of the livestock keepers have reformed their habits of consuming dead animals and raw livestock products such as milk, blood, and meat. Changes were due to the government education campaign in the last RVF outbreak intervention where the communities were educated about health risks of using raw livestock products ((Key informants). The government authorities-imposed restriction on eating of dead, sick animals and raw livestock products so as to reduce the risks of contracting RVF (Key informants). Despite education, some of the pastoralists continue with consumption of raw meat, milk and fresh blood on believing that they will be healthy and strong (Key informants). Some of the respondents believe that raw products contain many nutrients compared to cooked ones, thus why they prefer them (FGD members).

#### Climatic factors associated with RVF

Most Key Informants mentioned high temperature and heavy rainfall as the major climatic factors associated with occurrence of Rift valley fever. In comparison to other villages that are not located along the rift valley, the villages along the rift valley experience extremely hot environments. For instance, it’s extremely hot in Engarasero and Pinyinyi villages (Key informants). According to participants of the Engarasero, mosquitoes remain an issue throughout the year in some villages in the rift valley ecosystem (FGD members). Additionally, villages in the Rift Valley region become flooded whenever the year’s heavy rains have fallen (Key informants).

## Discussion

The aim of our study was to assess the knowledge, attitude and practices toward RVF disease. In this present study, a total of 352 people aged 18–65 years were interviewed, with the majority being men (67.61%). This result was probably due to Masai customs where women are not allowed to give information without permission of the heads of household [26]. Consequently, women hesitated at taking part in this study. As a result, our study has a higher proportion of male respondents than a comparable study conducted in Kenya by Abdi et al. [22], who reported that only 53.06% of respondents were men. Moreover, the majority of participants were found to be at an age group of 25–34 years old. This result was attributed by voluntary participation of this age group in the study compared to other age groups (Researcher’s observation).

Similarly, the study found that majority (39.5%) of respondents had primary education and 58.2% were pastoralists. It is known that pastoralist tribes send children to primary school or not at all in order to take care of livestock or to protect their cultures [26]. Only few individuals allowed to join secondary school and higher studies. These results are based on the history of Masai and Sonjo tribes whose origin is livestock keeping. These are ethnic tribes inhabited the Ngorongoro district in high percentages [26]. Our study populations had lower educational levels than a similar study done in Uganda; approximately 12% of our participants had secondary educations, compared to 55% of participants in Uganda [27]. In comparison to a study by Abdi et al. [22], in Kenya, our study identified a lower proportion (36.6%) of respondents with informal education compared to a higher proportion (88%) of respondents who received informal education in Kenya.

Dissemination of information about diseases among the communities is crucial for safeguarding the public’s health. According to our findings, the majority of the participants in Ngorongoro had heard of RVF. This was not unexpected as the Ngorongoro district had experienced several RVF epidemics between 1947 to 1978, and from 1997 to 2007 [2,18]. The majority of the participants reported to get information about diseases through radio and calls from friends or family members and other livestock keepers from Kenya. Sometimes they get information from the community meetings and government officials. These results are probably due to the fact that many agropastoral and pastoralists in the study area live in environments that lack a reliable source of electricity. Lack of expertise in livestock and agriculture sectors in some villages of Ngorongoro district can contribute to poor dissemination of information to the communities (Researcher’s observations). Additionally, inadequate facilitation from the government may lead to insufficient dissemination of health education among the communities.

The assessment of knowledge and attitude among communities about mosquito-borne infections is important in planning management strategies. This study revealed that 36.1% and 76.42% of participants had good knowledge and positive attitudes on mosquito-borne diseases, respectively. Whereas the majority (89.22%) of them identified malaria as the only disease spread by mosquito bites. These results were probably due to various initiatives campaigns to eradicate malaria carried out here in Tanzania [28]. For instance, the Minister of Health announced the following: “our responsibility is to ensure that the prevalence of malaria decreases up to zero percent. For this reason, we must have a continuous exercise of distributing long-lasting insecticides treated bed nets to all households in Tanzania. Similarly, we must use health centers to reach the primary beneficiaries: mothers who attends clinics and children under one year old" [28]. Our study revealed the benefits of government’s initiatives because majority (88.35%) of participants reported the use of mosquito nets. This finding was probably due to health education on prevention of malaria provided to the communities by the health workers.

Knowledge about the modes of transmission of RVF infection is critical for executing proper deterrent measures and thereby lowering the likelihood and magnitude of RVF epidemics. The majority of respondents surveyed in this study were unaware of mosquitoes’ role as the principal vectors for transmission of RVF virus to animals. Only 13.64% of respondents knew that RVF virus is transmitted through mosquito bites, in spite of believing that mosquitoes have adverse effects to livelihood. The majority of them reported malaria as the major mosquito-borne disease. These findings are almost parallel to those reported in studies conducted in Tanzania [16], Kenya [22] and Democratic Republic of Congo [29].

Correspondingly, this study evaluated the general knowledge, attitude and practices toward RVF among agropastoral and pastoral communities. The study found that 36.1%, 38.64% and 16.19% of participants had good knowledge, positive attitude and effective infections preventive practices toward RVF, respectively. Generally, these results indicated that the studied communities had poor knowledge, negative attitude and non-effective infection preventive practices toward RVF disease. Similar results were found in various studies done in Africa where agropastoral and pastoral societies found to have low KAP regarding RVF occurrences [16,22,30,31,32,33] The lack of education may have influence on KAP of the communities toward interventions that could reduce risks of RVF virus transmission and RVF epidemics.

Despite the fact that 52% of participants had heard of RVF, the majority were unable to identify the most significant signs of the disease in both humans and animals. Fever (increased body temperature) was the most prevalent symptom reported in both humans and animals. Regarding the signs of RVF in humans, unlike fever and headache, other symptoms (muscle pain, joint pain, backache, blurred vision and lethargy) were quantified by less than 11% of the participants. The outcomes of our study differ from the study by Abdi et al. [22] in Kenya, who reported that the majority (92%) of respondents identified hemorrhage as the key sign of RVF in humans and other signs by less than 40%. These findings suggest that most of participants are unaware of how RVF manifests clinically in humans. This was probably due to lack of regular educational campaigns about RVF in some villages in Ngorongoro district.

Despite the fact that Ngorongoro district had experienced numerous outbreaks of RVF, this study showed that the communities had poor knowledge regarding RVF clinical signs in animals. Apart from hemorrhagic fever, abortion and fetid diarrhea other signs were pronounced by less than 8% of participants. This was in contrast to research conducted among livestock keepers in Tanzania (poor awareness of RVF clinical indications was reported with a score of less than 50%) and in Kenya (knowledge score of less than 25%) [22,32]. This indicates that people forget what they learn about RVF from earlier epidemics as time passes and this reinforces the need for consistent and continued education and sensitization of communities outside of active outbreaks.

Furthermore, the findings revealed that only around 8% of the communities’ members were aware that consumption of raw meat and milk, direct contact with contaminated surfaces or handling of aborted fetuses on bare hands, and sheltering animals in their houses are all risk factors for RVF transmission. The results of this study, which showed that raw livestock products are consumed by the highest proportion, reflect findings from previous research conducted in Kenya [22, 32, 33, 34], Uganda [27] and Tanzania [16]. The study conducted in Kenya documented that pastoralist communities shelter livestock in human houses and drink raw milk, regardless of health education provided during RVF epidemics [22,32,34]. These risk factors increase the likelihood of the communities in Ngorongoro district and their livestock to get RVF infections and other zoonoses. Therefore, the government should keep on educating the communities on risks of consuming raw livestock products and use of protective gears during handling of sick or dead animals.

According to this survey, the majority of respondents do not consider Rift Valley Fever to be a dangerous disease of public health concern, despite the fact that RVF outbreaks are common in Ngorongoro district. Also, they don’t believe that they are at a high risk of contracting RVF. Moreover, the majority of them do not feel that wearing protective gears during the environmental cleanliness will protect them from catching RVF. Lastly, despite the existence of wild animals in Ngorongoro district, the majority of respondents do not believe that interactions among humans, domestic, and wild animals can facilitate the spread of the RVF virus. The lack of communities’ awareness about the disease was linked to the participants’ negative attitudes in this study (P = 0.000). This suggests that health education should be a continuous practice in agropastoral and pastoral communities in order to prevent the occurrence of RVF infection. These outcomes correspond well with findings reported in various surveys conducted worldwide, in which communities expressed a negative attitude towards RVF transmission [22,31].

The recent study indicated that general knowledge about RVF is not related with age, marital status and religion but significantly related to sex, education and locality. Males were more likely aware of RVF compared to the females (P = 0.04). This is probably connected with socio-cultural practices of Maasai people, where males are mostly involved in animals’ diseases management compared to the females. The finding of this study differs from those reported by Abdi et al. [22], in Kenya, whereby knowledge was not linked to sex. Also, individuals with formal education (primary, secondary and college) were found to be more knowledgeable on RVF than informal ones (P<0.05). This result implies that level of knowledge can in fact be influenced by level of education. Similar results were documented by Owange et al. [10] in Kenya where, respondents with tertiary education were more knowledgeable on RVF. Lastly, the residents of Pinynyi and Sale villages demonstrated good practices of RVF prevention compared to those of Orgosorok (P<0.05). This finding is attributed by a well-organized health delivery system in these villages (Key Informants in Pinyinyi and Sale). The community health workers usually conduct a monthly field visit for the purpose of health education deliverance in their communities (Key Informants in Pinyinyi). Therefore, improving publics’ education will lead into satisfactory knowledge which in turn will enhance good practices.

Moreover, our study revealed that during the last RVF outbreak in 2007, the government conducted vaccination campaign to the small ruminants. Unfortunately, the intervention came after livestock had already developed RVF infection. Since vaccination was done late, the livestock keepers did not see good recovery impact as their livestock continued to die. Therefore, the livestock keepers advised that the vaccine should be delivered before the RVF manifests (between December and June). Additionally, the government continued to vaccinate sheep and goats in 2011 and 2012 even though there were no RVF cases. After that, no further vaccination took place in Ngorongoro, so the livestock keepers stopped vaccinating their livestock. The government was graciously requested by the communities to enhance vaccination initiatives through the private sector so as to prevent RVF epidemics from recurrences in Ngorongoro and the country as a whole.

Lastly, this study found that most of the Key Informants were aware of climatic factors associated with RVF occurrences. Most of the them were managed to associate the last epidemic in 2007 with high temperature and heavy rainfall. They defined that the villages along the Rift valley ecosystem were heavily flooded and there was massive death of livestock during the outbreak. The previous studies conducted in Kenya [22,32,33] and Tanzania [2,16,18] had linked these factors with the RVF epidemics in the eastern ecosystem of Rift Valley. The eastern Rift Valley ecosystem is characterized by bimodal rainfall patterns which give suitable environment for growth and survival of vectors [2,18].

### Limitations of the study

Because this study was conducted 14 years since the last RVF epidemic in 2007, the results may have been influenced by failure to recall phenomena. The KAP survey was established to collect baseline information in order to inform the government of the current situation in RVF-prone areas. Finally, because the participants were questioned about their knowledge, attitudes, and practices regarding RVF, there may be bias in responses based on how participants wish to fit in the KAP.

### Conclusions and recommendations

According to a recent survey conducted in Ngorongoro district, agropastoral and pastoral residents have inadequate knowledge, unfavorable attitudes, and unsatisfactory practices when it comes to RVF. The recommended preventive measures toward RVF are not followed by these communities. Therefore, the awareness and practices regarding RVF epidemiology needs to be improved so that these communities can protect themselves and their livestock from recurrences of epidemics. Well-planned education campaigns on livestock vaccination and mosquito control would be effective methods to help prevent the spread of RVF. Phone and radio-based information dissemination must be well thought out, as phone and radio have been found to be the most widely used modes for communication. For instance, developing a phone application with the aim of educating the communities on RVF transmission, symptoms and management, could be an easily accessible way to continue to deliver consistent education and sensitization to communities. Health education should be continued and strengthened when there is a high chance of RVF outbreak. Finally, the government should ensure that personal protective gears are available to the agropastoral and pastoral communities, and strongly promote their usage.

## Supporting information

S1 Table Knowledge about Mosquito-borne zoonoses and control measures in Ngorongoro district. (DOCX)

S2 Table The knowledge of agropastoral and pastoral communities about RVF signs and transmission in Ngorongoro district, Tanzania. (DOCX)

S3 Table Proportion of communities’ attitude toward Mosquitoes borne diseases. (DOCX)

S4 Table Proportion of communities’ attitude toward RVF transmission and prevention. (DOCX)

S5 Table Proportions of communities’ responses on practices about RVF prevention in Ngorongoro district. (DOCX)

S1 Data Excel files of general RVF KAP data collected from agropastoral and pastoral communities of Ngorongoro district. (XLSX)
