# Supplementary material for: Knowledge, attitudes and practices on rift valley fever among pastoral and agropastoral communities of Ngorongoro in the rift valley ecosystem, Tanzania, conducted in 2021/2022
Source: PLoS Negl Trop Dis. 2023 Aug 23;17(8):e0011560. doi: 10.1371/journal.pntd.0011560 (PMC10479901; doi:10.1371/journal.pntd.0011560)
Supplement: S1 Table — (DOCX) [file pntd.0011560.s001.docx]

**S1 Table:** **Knowledge about Mosquito-borne zoonoses and control measures in Ngorongoro district**

| Variable | Frequency (n) | Proportion (%) |
| --- | --- | --- |
| Know mosquitoes | **n=352** |  |
| Yes | 349 | 99.15 |
| No | 3 | 0.85 |
|  |  |  |
| How often have you bitten by mosquitoes | **n=352** |  |
| Everyday | 263 | 74.72 |
| Rarely | 54 | 15.34 |
| I don’t know | 35 | 9.94 |
|  |  |  |
| What effects do Mosquitoes have to the quality of your life? | **n=352** |  |
| Transmit pathogen | 307 | 87.23 |
| Cause nuisance | 24 | 6.82 |
| I don’t know | 21 | 5.97 |
| Mosquito-borne diseases known/mentioned | **n=353*** |  |
| Malaria | 314 | 89.22 |
| Dengue | 1 | 0.03 |
| I don’t know | 38 | 10.79 |
|  |  |  |
| Species of mosquitoes known/mentioned | **n=364*** |  |
| Aedes | 2 | 0.57 |
| Anopheles | 77 | 21.88 |
| Culex | 12 | 3.41 |
| I don’t know | 273 | 77.56 |
|  |  |  |
| Which measures do you use to reduce mosquito population? | **n=361*** |  |
| Use of insecticides sprays | 22 | 6.25 |
| Eliminate stagnant water | 5 | 1.42 |
| Bush clearance | 71 | 20.17 |
| I do nothing | 256 | 72.72 |
| Kill as noticed | 7 | 1.99 |
|  |  |  |
| Which control measure do you take to protect yourself and your family from being bitten by mosquitoes? | **n=355*** |  |
| Mosquito netting | 311 | 88.35 |
| Repellents | 3 | 0.85 |
| Stay indoor | 11 | 3.13 |
| I don’t know | 30 | 8.52 |
|  |  |  |
| Which season of the year mosquito population is high? | **n=352** |  |
| Rain season | 335 | 95.17 |
| I don’t know | 17 | 4.83 |

****Respondents gave multiple response***
