# Supplementary material for: Knowledge, attitudes and practices on rift valley fever among pastoral and agropastoral communities of Ngorongoro in the rift valley ecosystem, Tanzania, conducted in 2021/2022
Source: PLoS Negl Trop Dis. 2023 Aug 23;17(8):e0011560. doi: 10.1371/journal.pntd.0011560 (PMC10479901; doi:10.1371/journal.pntd.0011560)
Supplement: S2 Table — (DOCX) [file pntd.0011560.s002.docx]

**S2 Table: The knowledge of agropastoral and pastoral communities about RVF signs and transmission in Ngorongoro district, Tanzania**

| Variable | Frequency (n) | Proportion (%) |
| --- | --- | --- |
| Have you heard about RVF disease? | **n=352** |  |
| Yes | 184 | 52.00 |
| No | 168 | 48.00 |
| Is the disease zoonotic? | **n=352** |  |
| Yes | 88 | 25.00 |
| No | 264 | 75.00 |
| Types of animals can be infected | **n=428*** |  |
| Humans | 71 | 20.17 |
| Ruminants | 161 | 45.74 |
| Wild animals | 9 | 2.25 |
| I don’t know | 187 | 53.13 |
| Common clinical signs of RVF in humans | **n=401*** |  |
| Hemorrhagic fever | 5 | 1.42 |
| Febrile fever | 47 | 13.35 |
| Muscle pain | 18 | 5.11 |
| Backache | 9 | 2.56 |
| Convulsion | 3 | 0.85 |
| Lethargy | 11 | 3.13 |
| Headache | 36 | 10.23 |
| Blurred vision | 5 | 1.42 |
| Joint pain | 15 | 4.26 |
| I don’t know | 282 | 80.11 |
| Common clinical signs of RVF in animals | **n=488*** |  |
| Hemorrhagic fever/fever | 107 | 30.40 |
| Fetid diarrhea | 45 | 12.78 |
| Lameness | 9 | 2.56 |
| Lacrimation | 15 | 4.26 |
| Abortion | 62 | 17.61 |
| Incoordination | 22 | 6.25 |
| Mucopurulent discharge | 22 | 6.25 |
| Sudden death of animal | 27 | 7.67 |
| I don’t know | 201 | 57.10 |
| Routes of transmission of RVFV to animals | **n=353*** |  |
| Blood feeding flies | 28 | 7.95 |
| Mosquitoes' bites | 48 | 13.64 |
| Ticks' bites | 32 | 9.09 |
| Direct contact with fluid or contaminated fomites | 16 | 4.55 |
| I don’t know | 229 | 65.06 |
| Routes of transmission of RVFV to humans | **n=373*** |  |
| Consumption of raw meat | 29 | 8.24 |
| Direct contact with fluid | 28 | 7.95 |
| Drinking raw milk | 20 | 5.68 |
| Mosquitoes' bite | 4 | 1.14 |
| Sheltering animals in house | 10 | 2.84 |
| I don’t know | 282 | 80.11 |

****Respondents gave multiple response***
