# Supplementary material for: Knowledge, attitudes and practices on rift valley fever among pastoral and agropastoral communities of Ngorongoro in the rift valley ecosystem, Tanzania, conducted in 2021/2022
Source: PLoS Negl Trop Dis. 2023 Aug 23;17(8):e0011560. doi: 10.1371/journal.pntd.0011560 (PMC10479901; doi:10.1371/journal.pntd.0011560)
Supplement: S3 Table — (DOCX) [file pntd.0011560.s003.docx]

**S3 Table: Proportion of communities’ attitude toward Mosquitoes borne diseases**

| **Variable** | **Number (%) of respondents’ responses** | | | | |
| --- | --- | --- | --- | --- | --- |
|  | **Completely agree** | **Agree** | **Neutral** | **Disagree** | **Completely**  **disagree** |
| Believing that mosquitoes have impact on their quality of life | 57(16) | 210(60) | 56(16) | 25(7) | 4(1) |
| Believing that they at risk of acquiring mosquito borne diseases | 28(8) | 202(57) | 64(18) | 34(10) | 24(7) |
| Believing that mosquitoes’ bites can transmit diseases | 40(11) | 221(63) | 67(19) | 19(6) | 5(1) |
| Always concerned with mosquitoes’ management | 19(5) | 218(62) | 59(17) | 48(14) | 8(2) |
