# Supplementary material for: Knowledge, attitudes and practices on rift valley fever among pastoral and agropastoral communities of Ngorongoro in the rift valley ecosystem, Tanzania, conducted in 2021/2022
Source: PLoS Negl Trop Dis. 2023 Aug 23;17(8):e0011560. doi: 10.1371/journal.pntd.0011560 (PMC10479901; doi:10.1371/journal.pntd.0011560)
Supplement: S4 Table — (DOCX) [file pntd.0011560.s004.docx]

**S4 Table: Proportion of communities’ attitude toward RVF transmission and prevention**

| **Variable** | **Number (%) of respondents’ responses** | | | | |
| --- | --- | --- | --- | --- | --- |
|  | **Completely agree** | **Agree** | **Neutral** | **Disagree** | **Completely**  **disagree** |
| Rift Valley Fever is a hazardous disease of public health importance | 22(6) | 83(24) | 172(49) | 71(20) | 4(1) |
| Humans are at high risk of being infected with RVF virus in this district | 27(8) | 73(21) | 132(37) | 99(28) | 21(6) |
| Spread of RVF virus infection to both humans and animals can be prevented | 41(12) | 124(35) | 143(41) | 37(10) | 7(2) |
| Vaccination of livestock against RFV is crucial. | 58(16) | 127(37) | 132(37) | 31(9) | 4(1) |
| Wearing protective gears while cleaning the environment prevents RVF transmission. | 25(7) | 86(24) | 147(42) | 64(18) | 30(9) |
| Proper vectors management can prevent RVF outbreak | 28(8) | 95(26) | 150(43) | 55(16) | 24(7) |
| Interaction between human, domestic, companion and wild animals can facilitate spreads of RVF virus. | 29(8) | 92(26) | 154(44) | 55(16) | 22(6) |
| Health workers can solve the problem of RVF outbreaks in this district. | 39(11) | 88(25) | 174(50) | 45(13) | 6(2) |
| You always provide information regarding sick or deceased animals | 40(12) | 114(32) | 103(29) | 89(25) | 6(2) |
