# Supplementary material for: Knowledge, attitudes and practices on rift valley fever among pastoral and agropastoral communities of Ngorongoro in the rift valley ecosystem, Tanzania, conducted in 2021/2022
Source: PLoS Negl Trop Dis. 2023 Aug 23;17(8):e0011560. doi: 10.1371/journal.pntd.0011560 (PMC10479901; doi:10.1371/journal.pntd.0011560)
Supplement: S5 Table — (DOCX) [file pntd.0011560.s005.docx]

**S5 Table**: **Proportions of communities’ responses on practices about RVF prevention in Ngorongoro district**

| Variable | Frequency (n) | Proportion (%) |
| --- | --- | --- |
| Avoid contact with blood/fluids from sick or dead animals |  |  |
| Yes | 16 | 4.55 |
| No | 336 | 95.45 |
| Avoid handling of aborted fetuses on bare hands |  |  |
| Yes | 16 | 4.55 |
| No | 336 | 95.45 |
| Eat well cooked meat from domestic and wildlife animals |  |  |
| Yes | 279 | 79.26 |
| No | 73 | 20.74 |
| Drink properly boiled milk |  |  |
| Yes | 269 | 76.42 |
| No | 83 | 23.58 |
| Eat wild animals |  |  |
| Yes | 113 | 32.29 |
| No | 237 | 67.71 |
| Drink fresh blood collected from animals |  |  |
| Yes | 212 | 60.23 |
| No | 140 | 39.77 |
| Wear protective gears during handling sick or dead animals |  |  |
| Yes | 6 | 1.71 |
| No | 346 | 98.29 |
| Avoid keeping animals inside their houses |  |  |
| Yes | 80 | 22.73 |
| No | 272 | 77.27 |
| Use of insecticides treated nets |  |  |
| Yes | 125 | 35.51 |
| No | 227 | 64.49 |
| Use of mosquito repellents |  |  |
| Yes | 21 | 5.97 |
| No | 331 | 94.03 |
| Treat ponds/stagnant water with insecticides |  |  |
| Yes | 6 | 1.71 |
| No | 346 | 98.29 |
